# Call for Biohistory Guidelines

**DOI:** 10.1371/journal.pmed.0020192

**Published:** 2005-06-28

**Authors:** Jordan Paradise

**Affiliations:** Chicago-Kent College of LawChicago, IllinoisUnited States of America

I am writing in response to an essay published in the most recent issue of *PLoS Medicine* by Deborah Hayden, entitled “Alas, Poor Yorick: Digging Up the Dead to Make Medical Diagnoses” [1]. As a co-author of the *Science* piece with Lori B. Andrews that Hayden references, I am troubled by her comment on our article. Nowhere in that article, “Ethics. Constructing Ethical Guidelines for Biohistory” [2], do we suggest that genetic testing be done on deceased individuals for historically significant questions. In fact, we specifically highlight some of the ethical, legal, social, and scientific issues that such testing raises and recommend that guidelines be developed in order to monitor current research that is being undertaken in this area. The article does not advocate biohistorical research. This distinction is very important and one that is quite evident upon a careful reading of our article.
